# Rationally engineered 3D-dendritic cell-like morphologies of LDH nanostructures using graphene-based core–shell structures

**DOI:** 10.1038/s41378-019-0114-x

**Published:** 2019-12-16

**Authors:** Karthik Kiran Sarigamala, Shobha Shukla, Alexander Struck, Sumit Saxena

**Affiliations:** 1 Centre for Research in Nanotechnology and Science, Indian Institute of Technology Bombay, Mumbai, MH 400076 India; 2 Nanostructures Engineering and Modeling Laboratory, Department of Metallurgical Engineering and Materials Science, Indian Institute of Technology Bombay, Mumbai, MH 400076 India; 3 Faculty of Technology and Bionics, Rhein-Waal University of Applied Sciences, 47533 Kleve, Germany

**Keywords:** Organic-inorganic nanostructures, Organic-inorganic nanostructures

## Abstract

Functionalization of graphene-based materials using chemical moieties not only modify the electronic structure of the underlying graphene but also enable in limited enhancement of targeted properties. Surface modification of graphene-based materials using other nanostructures enhances the effective properties by minimally modifying the properties of pristine graphene backbone. In this pursuit, we have synthesized bio-inspired hierarchical nanostructures based on Ni–Co layered double hydroxide on reduced graphene oxide core–shells using template based wet chemical approach. The material synthesized have been characterized structurally and electrochemically. The fabricated dendritic morphology of the composite delivers a high specific capacity of 1056 Cg^−1^. A cost effective solid state hybrid supercapacitor device was also fabricated using the synthesized electrode material which shows excellent performance with high energy density and fast charging capability.

## Supercapacitors: morphology-driven performance

A nanostructure consisting of Ni-Co layered double hydroxide dendrites on a reduced graphene oxide shell offers a high specific capacity for energy devices. Transition metal layered double hydroxides are attractive electrode materials for energy storage devices. However, slow ionic diffusion and poor electrical conductivity limits their use. Now, an international team from India and Germany, led by Sumit Saxena, demonstrate the synthesis of Ni-Co layered double hydroxide dendrites grown radially on reduced graphene oxide shells. This morphology creates a large a number of active sites, enabling a high specific capacity of 1056 Cg^−1^. They also fabricate a solid state hybrid supercapacitor with their material as the cathode, achieving an energy density of 35 Wh kg^−1^ with fast charging. Further tuning of the morphology of the material may enable higher performance.

## Introduction

The increasing demand for non-conventional energy generation and storage has led to urgent need for exploration of novel materials systems and morphologies^1–7^. Transition metal layered double hydroxides (TMLDHs)^8^ show excellent electro-activity, have flexible ion exchange property, possess good redox activity, incur low costs, are environment friendly in nature and hence potential candidates for energy storage^9^. Recently, TMLDH’s with a generic formula represented by M^2+^_1−*x*_M^3+^_*x*_(OH)_2_·A^*n*−^_*x*/*n*_·zH_2_O, where M^2+^ and M^3+^ may be bivalent or trivalent metal cations, while A^n−^ is charge-balancing anion residing in the inter-lamellar region and *x* = M^3+^/(M^2+^ + M^3+^), are widely explored nowadays in both scientific research and industrial communities because of their applications in energy storage, catalysis, drug delivery and flame retardants to name a few^10,11^. The major disadvantage of these materials is that they enable slow ion diffusion and have poor electrical conductivity. This leads to poor cycle life, moderate charge–discharge rates and low rate capability^12^. In order to mitigate these issues several strategies are being explored. One way is to fabricate low-dimensional nanostructured transition metal oxides and incorporate with diverse carbon-based materials, such as carbon nanotubes, graphene, amorphous carbon^13–18^. It is interesting to note that the rate capability of most transition metal oxide/hydroxide-based electrodes can be remarkably improved after decorating with different carbon materials^19^. Their cycling stabilities unfortunately have not been improved. Recently carbon-based core–shell nanostructures are one of the most extensively explored materials for hybrid nanostructures^20^. Even though carbon core–shell-based materials have been explored for lithium-ion batteries, their application in supercapacitors is limited^21,22^. 3D core–shell nanostructured NiCo-LDH@CNTs carbon as electrode materials for supercapacitors exhibit a high specific capacitance of 1023 Cg^−1^ at 1 Ag^−1^due its unique structural design, good electrical conductivity and large specific surface area^23^. Ni-Al LDH/CNT core–shell nanostructures have high surface area and exhibit a specific capacity of 1071 Cg^−1^ at a current density of 0.5 Ag^−1^ ^24^. NiCo_2_O_4_ nanowires grown on carbon fiber paper also demonstrate enhanced electrochemical performance^25^. Of all carbon-based materials, graphene stands out due to large surface area, excellent conductivity, good chemical stability and easy processability^26^. These extraordinary properties have enabled exploration of graphene as an electrode material in batteries, solar cells and supercapacitors to name a few^27^. Nanocomposites derived from most of the conventional processes end up in vertically stacked self-assembly of LDHs intercalated in graphene sheets^28,29^. Therefore, limited access to effective surface area and low porosity in such nanocomposites act as bottleneck in utilizing the true potential of these nanocomposites. These problems can be mitigated by adopting graphene-based hierarchical core–shell morphologies at nanoscale. Nanostructures of rGO with LDHs are expected to provide optimal solution for charge storage applications^30^. LDH lamellae which are radially self-assembled over the rGO core structures show enhanced electrochemical performances due to good electrical conductivity, high specific surface area and good stability. They provide large number of active sites for participating in the redox process to obtain high specific capacitance. These surface engineered structures are morphologically represented by numerous membrane like structures extending out from the core of rGO. Such morphologies ensure large effective surface area, a conducting core and an unconstrained ionic transport within the porous channels of the electrode material. Synthesis of such rationally designed nanostructures is a challenge.

In order to achieve this, we prepare graphene oxide encapsulated silica nanoparticles. During the synthesis process rGO skeletal structure is formed by elimination of silica particles. This yields a unique 3D structure with multiple open channels that inhibits the agglomeration of Ni–Co LDH nanosheets facilitating the formation of the homogeneous dendritic cell-like morphologies (Ni–Co LDH@rGO) for dispersal in solution. They provide a large number of active sites for participation in redox processes as compared to traditionally synthesized composites. These rationally designed hybrid nanostructures not only greatly enhance the electrical conductivity but also reduce low diffusion resistance to ionic species. This results in a synergetic effect which facilitates efficient energy storage at high rates as compared to traditionally synthesized Ni–Co LDHs. The as synthesized Ni–Co LDH@rGO delivers a high specific capacity of 1056 Cg^−1^. Further performance of solid state hybrid supercapacitor fabricated using synthesized material is demonstrated. The device shows excellent performances with a high energy density of 35 Whkg^−1^ with fast charging. This shows that the synthesized material provides a good platform to synthesize structural assembly of tuneable materials with huge potential in highly advanced electrodes for supercapacitor application.

## Materials and methods

### Preparation of GO wrapped SiO_2_ nanospheres (SG)

Initially 0.4 g of synthesized SiO_2_ spheres were taken and dispersed in 25 ml of DMF solution and sonicated for an hour. Subsequently 1.3 ml of APTES was added to the above mixture and vigorously stirred at 110 °C for 2 h. Then 100 mg of GO solution with a desired concentration of 1 mg/ml is dispersed into the above solution. 1.3 g of DCC is now added to the above suspension and stirred uniformly. After 24 h the resultant mixture is cleaned with de-ionized water using centrifugation and dried at 60 °C to obtain SiO_2_/GO (SG).

### Preparation of dendritic cell-like structures of Ni–Co LDH@rGO

Ni–Co LDH@rGO composite is synthesized by initially adding 30 mg of SG spheres to 25 ml of aqueous alcoholic emulsion and the mixture is sonicated for 30 min. Ni(NO_3_)_2_.6H_2_O and Co(NO_3_)_2_.6H_2_O (feeding ratio of 6:4 mol) and 0.28 g of HMT were added to the above solution and sonicated. Subsequently the homogeneous mixture was transferred into a teflon lined autoclave vessel and heated for 12 h at 90 °C. The resultant product was washed thoroughly with ethanol and deionized water mixture several times. The final product is obtained after drying in vacuum at 60 °C overnight. For comparison pristine Ni–Co LDH was synthesized similarly but without SG nanospheres.

### Material characterizations and electrochemical measurements

The crystal lattice structure of the synthesized material is investigated by powdered X-ray diffraction (XRD) technique (PanalyticalX’Pert Pro using Kα irradiation with a wavelength of 0.1542 nm) at a scan speed of 4°min^−1^. The functional groups are identified using Fourier Transform Infrared (FTIR) spectroscopy (3000 Hyperion Microscope with Vertex 80 FTIR System, Bruker). The surface morphologies and nanostructures of the synthesized materials is characterized using a Field-Emission Scanning Electron Microscope (FEG-SEM, JEOL JSM-7600F FEG-SEM) and a High-Resolution Transmission Electron Microscope (HR-TEM) equipped with an Energy Dispersive X-ray spectroscopy (EDX). The vibrational modes of the materials are probed by Raman spectroscopy (Witec 300 RAS). Thermogravimetric analysis (TGA) of samples is performed using TGA Q500, (TA instrument).

Cyclic voltammetry (CV) and galvanostatic charge-discharge (GCD)measurements are performed in a three electrode configuration using Biologic SP-300. The working electrodes (Ni–Co LDH, Ni–Co LDH@rGO)are fabricated using a mixture of 80 wt% of the active material, 10 wt% of polyvinylidene difluoride (PVDF) and 10 wt% of carbon black. Ethanol is used as solvent. The prepared electrode slurry is pasted on Ni substrate and dried in vacuum at 80 °C. 3 M aqueous KOH is used as electrolyte. The mass loadings of the electrode is about 4 mg.

### Device assembly

Two electrode hybrid device with positive electrode as Ni–Co LDH@rGO and negative electrode as rGO is prepared. The electrode slurry is coated uniformly on an inexpensive graphite foils with an area of 2 × 2 cm^2^. A hybrid device is assembled by sandwiching polymer electrolyte (PVA/KOH electrolyte prepared with a wt/volume ratio of 2:3) in between both the positive and negative electrodes. Electrical contacts are drawn from both the current collectors using copper foils. Finally, the assembled device is sealed air tight.

## Results and discussion

### Composition and morphology of hybrid structures

**Fig. 1:**
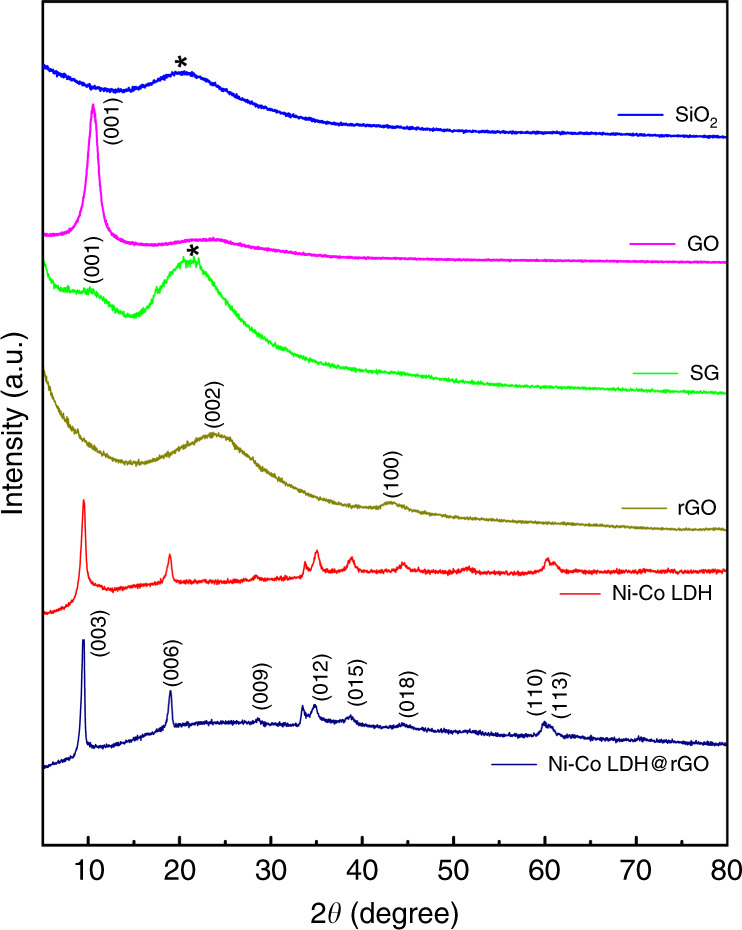
X-ray diffraction pattern of SiO_2_, GO, SG (SiO_2_/GO) core–shell structure, rGO, Ni–Co LDH, and Ni–Co LDH@rGO.

The crystal structure of the prepared samples is examined using powdered X-ray diffraction. Figure 1 shows the XRD pattern of silica. The broad hump suggest amorphous nature of the synthesized SiO_2_ nanospheres. The bragg’s peaks of exfoliated graphene oxide (GO) is observed at 10.4° which corresponds to (001) plane^31^. The presence of oxygen functional groups on the basal planes, as well as on the edges results in the negatively charged GO sheets.

These GO sheets are coated on the surface modified SiO_2_ spheres due to the electrostatic interaction. This finally results in formation of GO wrapped silica spheres (SG)^32^. This is seen as amorphous peak of silica along with a broad peak of GO. The XRD pattern of reduced graphene oxide (rGO) in the Fig. 1 shows a broad reflection at 24.7° confirming few layer graphene sheets and a low intense peak around 44° shows a turbostratic behavior of disordered carbon layers after the removal of oxygen containing function functional groups^33^. The Ni–Co LDH@rGO shows well-defined diffraction peaks corresponding to (003), (006), (009), (012), (015), (018), (110), and (113) plane. These bragg’s reflections correspond to hydrotalcite like LDH phase and with an average interplanar spacing (d_003_) of 9.28 Å. The unit cell parameters are calculated using the XRD data. It is seen that both the pristine and the composite samples have nearly the same values of ‘a’ and ‘c’ lattice constants. This suggests that the composite retains structural features of the LDH and is reflected in both the metal–metal distance in the brucite–like layers and the interlayer distance^34,35^. The bragg’s reflections observed in X-Ray diffraction pattern for Ni–Co LDH and Ni–Co LDH@rGO are also identified in the diffraction fringes are shown in the supplementary Fig. S1. The polycrystalline nature of the nanostructured samples also aid in the charge storage capability due to its enhanced surfaced area and the availability of active sites for diffusion of ions^36^.

**Fig. 2:**
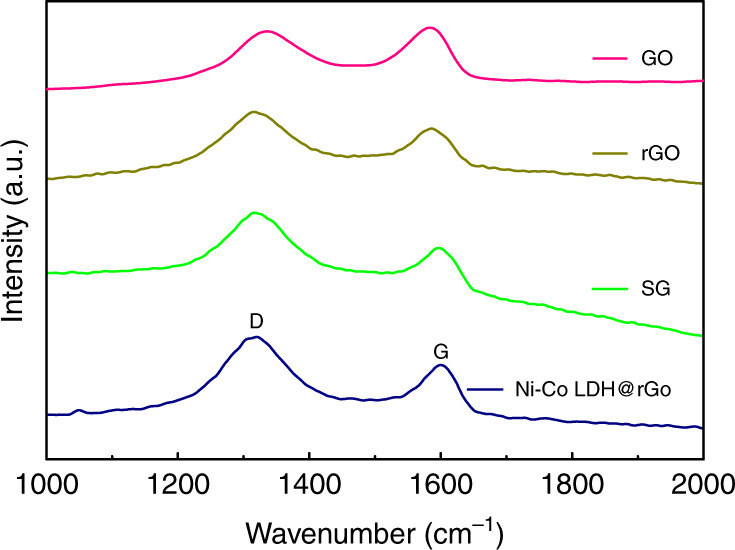
Raman spectra recorded for GO, rGO, Ni–Co LDH@rGO showing D and G bands corresponding present in graphitic systems.

Raman spectroscopy is used to complement the XRD data for identification of rGO in Ni–Co LDH@rGO, to study disorderedness and crystal lattice defects. Figure 2 shows representative the Raman spectra for GO, rGO, SG, and Ni–Co LDH@rGO. The structural defects and the edges that impair the translational symmetry results in ‘D’ band while the ‘G’ band denotes the first-order scattering of E_2g_ phonons^37^. The ‘D’ band occurs at approximately 1330–1340 cm^−1^ while the Raman allowed ‘G’ band is observed at around 1580–1600 cm^−1^. This is observed in SP^2^ carbon systems and arises from C–C bond stretch formed from first order Raman scattering. The peak intensity ratio (I_D_/I_G_) is used to quantify the degree of graphitization of carbon materials. The peak intensity ratio (I_D_/I_G_) of Ni–Co LDH@rGO (1.3) is higher than the rest SG(1.19), rGO(1.12), GO(0.98). This suggests that the Ni–Co LDH@rGO has a large number of defects in rGO. These defects on the surface of rGO are vital in supporting the nucleation process and inhibiting agglomeration of the Ni–Co LDH nanocrystallites during the growth process.

**Fig. 3:**
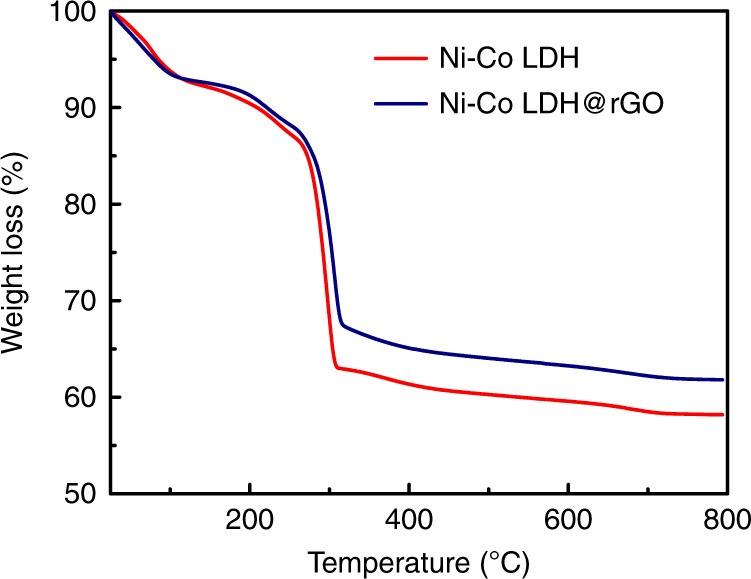
TGA profiles for the pristine Ni–Co LDH and Ni–Co LDH@rGO.

TGA is used to analyze the thermal stability of the pristine Ni–Co LDH and Ni–Co LDH@rGO. The thermal decomposition pattern of the Ni–Co LDH is typically ascribed to water loss (both free and physisorbed) followed by dehydroxylation^38^. In the TGA pattern of the Ni–Co LDH@rGO nanohybrids shown in Fig. 3, three major weight loss stages are observed^39–41^. The first weight loss component occurs at ~120 °C and is attributed to the removal of loosely bound water molecules from the interlayers. The second weight loss component in the temperature range from 200–320 °C is understood to occur due to the removal of oxygen functionalities. The third and final weight loss component, observed in the temperature above 400 °C is primarily due to the de-hydroxylation and de-carbonation of the nanosheets. The difference of 4–5% weight loss stage between 350 and 750 °C can be ascribed to the decomposition of rGO, the weight loss percentage of rGO in Ni–Co LDH@rGO. The weight loss percentage between 350 and 750 °C for Ni–Co LDH@rGO composite is about 38% and for the pure sample is 42%. The steady weight loss difference around 600 °C is attributed to the loss of adsorbed water, residual and oxygenated functionalities. The elimination of thermally imbalanced oxygen functional groups results in a thermally stable material. A high thermal stability is retained in Ni–Co LDH@rGO due to the presence of rGO. The results show that the nanohybrids have characteristic traits of both pristine LDH, as well as rGO.

**Fig. 4:**
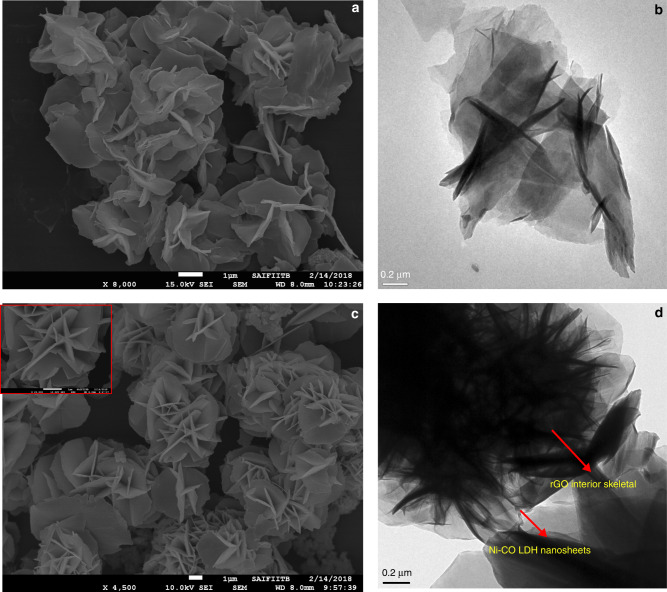
Surface morphological studies of synthesized materials using electron microscopy. (**a**) SEM micrograph of Ni–Co LDH, (**b**) HR-TEM micrographs of Ni–Co LDH; (**c**) SEM micrograph of Ni–Co LDH@rGOand (**d**) HR-TEM image of Ni–Co LDH@rGO.

The surface morphologies of the Ni–Co LDH and Ni–Co LDH@rGO composites are investigated using electron microscopy. The SEM micrographs of pristine Ni–Co LDH in Fig. 4a suggests formation of aggregated compact lamellar sheets. The agglomeration of nanosheets can also be clearly seen from the HR-TEM image as shown in Fig. 4b. In order to inhibit such agglomerated structures, it is essential to synthesize nanostructures that support LDH’s to build robust and flexible architecture by self-assemblies. A 3D hybrid composite with spherical structure is obtained by self-assembly of LDH nanosheets on the graphene oxide shells. This is observed for Ni–Co LDH@rGO composite, shown in the Fig. 4c.

A large number of evenly and well aligned thin LDH lamellae self-assemble radially on the surface of the graphene structures in a 3D unstacked porous geometry. The vertically aligned channels are also seen in the magnified image as an inset in Fig. 4c. Such 3D structures provides hierarchical porous features with wide distribution. The internal morphology of the Ni–Co LDH@rGO sample is imaged using HR-TEM images. It is evident in Fig. 4d that after the elimination of SiO_2_ template (HR-TEM images of GO coated SiO_2_ core–shell is shown in supplementary Fig. S2), a double skeletal structure with a 3D flower-like architecture is formed and the resultant morphology of the hybrid restricts aggregation of the LDH nanosheets. It is understood that the surface of graphene oxide shell acts as a confinement region and aids in self-assembly of Ni–Co LDH nanosheets radially outwards due to negatively charged GO sheets for continuous growth of LDH. Besides the conductive graphene unveils a high surface area for the ultrathin LDH nanosheets growth with uncluttered porous channels. Further it also serves as a 3D scaffold for the growth of dendritic cell-like morphology. The image also clearly illustrates fine and thin LDH lamellae self-assemble radially outwards over the rGO scaffolds. The average thickness of the obtained LDH nanosheets grown on the rGO skeletal structure is about 5−6 nm. The obtained LDH lamellae with huge surface area and hierarchical morphology facilitates the electrolyte transport and increases the electrochemical active sites effectively.

**Fig. 5:**
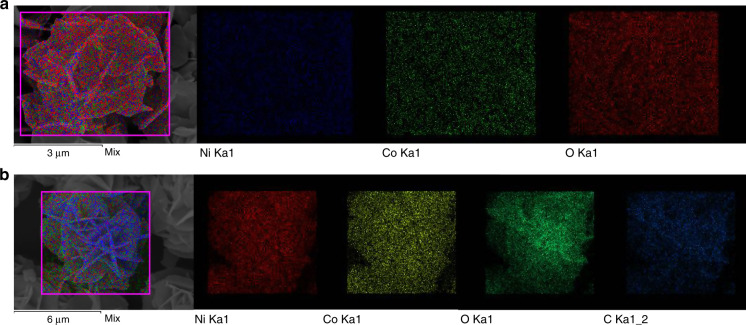
Elemental mappings recorded for (**a**) Ni–Co LDH and (**b**) Ni–Co LDH@rGO.

Presence of rGO in the Ni–Co LDH @rGO sample is verified using EDAX analysis and elemental mapping using FEG-SEM. The presence of carbon in the EDAX profiles is shown in supplementary Fig. S3. The elemental mapping shown in Fig. 5 indicate presence of rGO.

A comparative of elemental mapping in the pristine Ni–Co LDH and Ni–Co LDH@rGO nanocompositeis shown in Fig. 5a, b), respectively. It shows that the elements Ni, Co, O, and C homogeneously spread over the dendritic cell-like structure. It is also inferred from the figure that rGO is uniformly distributed and attached to the LDH surface in a skeletal shell like morphology, indicating a successful growth of the nanosheets. The STEM micrographs of the samples are provided as Supplementary Fig. S4a, b. It can be concluded that the synthesized hybrid material has a 3D structure with LDH lamellae self-assembled radially outwards over the reduced graphene oxide hold structural integrity and retain the spherical morphology.

### Electrochemical performance

**Fig. 6:**
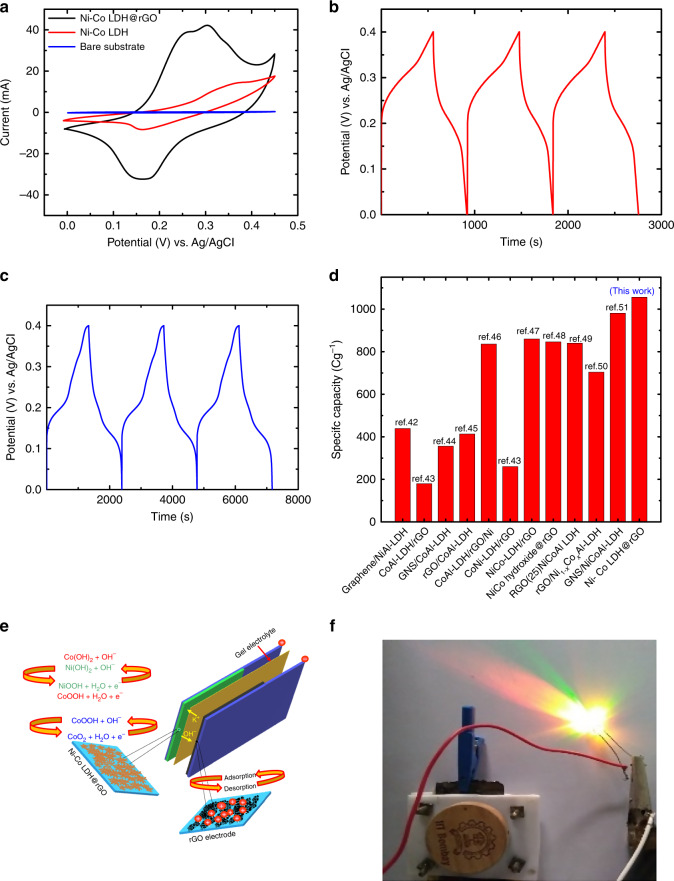
Electrochemical evaluation of the synthesized materials. **a** Shows cyclic voltammograms recorded at a scan rate of 5 mVs^−1^for Ni–Co LDH, Ni–Co LDH@rGO and bare substrate. **b** Galvanostatic charge-discharge curves for Ni–Co LDH at 1 Ag^−1^. **c** Galvanostatic charge-discharge curves for Ni–Co LDH@rGO at 1 Ag^−1^, (**d**) Comparison of specific capacity of Ni–Co LDH@rGO with other LDH-graphene composites available from literature (**e**) Device assembly and charge storage mechanism. **f** Prototype showing lighting a multicolor LED light with 3.5 V.

Electrochemical investigations are performed on pristine Ni–Co LDH and Ni–Co LDH@rGO in a three electrode cell in 3 M KOH using a Platinum counter electrode (1 × 1 cm^2^) and Ag/AgCl as a reference electrode. Figure 6a shows a comparative CV for Ni–Co LDH and Ni–Co LDH@rGO at 5 mVs^−1^. Both materials exhibit a pair of strong redox peaks in their CV curves, suggesting that the specific capacitance is primarily due to Faradaic redox reactions. In general, the area of CV curve is directly proportional to its specific capacitance. Evidently, the integral area under the CV curve and the current density of Ni–Co LDH@rGO is much larger than that of pristine Ni–Co LDH, which is suggestive of its higher specific capacitance. The superior performance of the composite electrode is ascribed to the synergistic effect of Ni–Co LDH nanosheets and highly conductive graphene core. Advantageously the radially aligned nanosheets on rGO core do not block the open porous channels thereby allowing charge storage on the graphene skeletal structure, as well as the LDH nanosheets from reaction with OH^−^ions. This signifies that the graphene core structure not only serve as a substrate but also acts as a spacer to inhibit the aggregation of Ni–Co LDH nanosheets and enhance the electrochemical performance. The impedance response of the synthesized materials is shown in Supplementary Fig. S6.

Figure 6b, c shows the GCD curves of Ni–Co LDH and Ni–Co LDH@rGO composite at a current density of 1 Ag^−1^ in the potential range of 0–0.4 V. GCD curves for 3000 cycles is shown in Supplementary Fig. S7. A divergent plateau region can be clearly seen which manifests a typical characteristics of Faradaic capacitance. This is also consistent with the CV profiles. The specific capacity is obtained from the GCD curves using the expression,

$$E_{{\mathrm{sc}}}\,=\,It{\mathrm{/}}m,$$

where *E*_sc_ is the specific capacity (Cg^−1^), *I* is the charge–discharge current (mA), *t* is the time (s) and *m* is the mass of the active material (mg). The specific capacitance obtained the for Ni–Co LDH and Ni–Co LDH@rGO composite are calculated to be 360 Cg^−1^ and 1056 Cg^−1^, respectively, at a current density of 1 Ag^−1^. Conspicuously, the specific capacitance is significantly enhanced after the growth of highly oriented fine LDH nanosheets with large number of active sites grown radially outwards on the graphene core structure. These 3D nanostructure offers a large surface area, short ion diffusion path and more efficient contact between the ions of the active material and the electrolyte thereby enhancing the specific capacitance. A comparative of the material performance with that of similar materials systems reported in literature^42–51^ is shown in Fig. 6d.

Further a solid state asymmetric supercapacitor device prototype is assembled to demonstrate practical application. The fabricated device configuration is shown in Fig. 6e. Ni–Co LDH@rGOis coated on a graphite sheet acts as positive electrode and rGO acts as a negative electrode. The two electrodes are separated by a thin alkaline-PVA solid gel electrolyte. This device is then assembled and tested for two electrode configuration.

A single device of Ni–Co LDH@rGO//rGO exhibits a high energy density of 35 Wh kg^−1^ and maximum power density of 3760 W kg^−1^ which are evaluated from the charge discharge profiles as shown in supplementary Fig. S5. The two such supercapacitor devices connected in series could light a multi-color light-emitting diode (LED) as shown in Fig. 6f. After charging at 10 mA for 30 s, the device could light the LED for over 15 min. The device photographs were taken at various intervals showing the intensity of light as a function of time shown in supplementary Fig. S8. The cycling performance of the assembled device for ~2500 cycles is shown in Supplementary Fig. S9. This shows that the fabricated hybrid supercapacitor has huge potential in various energy storage applications.

## Conclusion

In summary, a rationally designed distinctive nano-architecture composed of Ni–Co LDH@rGO dendritic cell-like morphology have been successfully synthesized using graphene as internal core–shell structure. These utilize synergistic contributions from both of the active materials Ni–Co LDH, graphene core and effectively integrate in a core/shell structure. Further, the advantages of the conductive and strong support of graphene scaffold structure enable the Ni–Co LDH electrode to deliver a high specific capacity of 1056 Cg^−1^ at a current density of 1 Ag^−1^. The as-assembled solid state hybrid supercapacitor device prototype (Ni–Co LDH@rGO//rGO) exhibits excellent performance with a high energy density of 35 Wh kg^−1^ and delivers a maximum power density of 3760 W kg^−1^.

## Supplementary information

Supplementary information
