# Supplementary material for: Rationally engineered 3D-dendritic cell-like morphologies of LDH nanostructures using graphene-based core–shell structures
Source: Microsyst Nanoeng. 2019 Dec 16;5:65. doi: 10.1038/s41378-019-0114-x (PMC8433191; doi:10.1038/s41378-019-0114-x)
Supplement: Supplementary file 1 — Supplementary information [file 41378_2019_114_MOESM1_ESM.docx]

**Supplementary Information**

**Rationally engineered 3D-dendritic cell like morphologies of LDH nanostructures using graphene based core shell structures**

Sarigamala Karthik Kiran^1^, Shobha Shukla^2^, Alexander Struck^3^ and Sumit Saxena^2*^

^1^ Centre for Research in Nanotechnology and Science, Indian Institute of Technology Bombay, Mumbai, MH, India - 400076.

^2^ Nanostructures Engineering and Modeling Laboratory, Department of Metallurgical Engineering and Materials Science, Indian Institute of Technology Bombay, Mumbai, MH, India - 400076.

^3^Faculty of Technology and Bionics, Rhein-Waal University of Applied Sciences, Kleve, Germany 47533.

^*^corresponding author: - sumit.saxena@iitb.ac.in

**Reagents and materials.** The reagents used in the synthesis were of analytical grade. Commercially available Ni(NO_3_)_2_.6H_2_O, Co(NO_3_)_2_.6H_2_O, graphite powder, P_2_O_5_, KMnO_4_, H_2_SO_4_, NaNO_3_, Tetraethyl orthosilicalte (TEOS), Ammonia, ethanol, hexamethylenetetramine (HMTA), Dimethyl formamide (DMF), N,N′-Dicyclohexylcarbodiimide (DCC), (3-Aminopropyl)triethoxysilane (APTES)

**Preparation of Graphene oxide**

Graphene oxide (GO) was prepared according to the modified hummers method^1^. Equal amounts of pre-oxidized graphite and NaNO_3_ are taken in 50mL sulfuric acid and the mixture is refluxed for an hour. To the above mixture 6gm of KMnO_4_ is slowly added for oxidation and the setup is maintained at 5°C. Then, after the oxidation process is completed add 80mL of de-ionized water with constant stirring for 30 min and the temperature of the mixture raised to 90°C. Next add deionized water followed by 6 ml H_2_O_2_ and stir the above solution for 1 hour. Finally, the pH of the solution is made neutral by washing it several times.

**Synthesis of SiO_2_ spheres**

SiO_2_ spheres are synthesized using modified stober’s method^2^. In a typical synthesis procedure 0.4 gm of CTAB was dissolved in an alcoholic emulsion containing 50 ml ethanol and 125 ml deionized water and 15 ml ammonia. The mixture was vigorously stirred for 30 minutes at 35˚C then 5 ml of TEOS was quickly dropped into the above solution. The resultant mixture was stirred for 5 hours and a white precipitate was obtained which is washed with deionized water several times to remove the surfactant.

**Figure S1:** Diffraction fringes with SAED patterns (inset) for (a) Ni-Co LDH (b) Ni-Co LDH@rGO

**Figure S2**: -Schematic showing GO coated SiO_2_ spheres prepared from SiO_2_ spheres and GO nanosheets

**Figure S3 :** EDAX profile of Ni-Co LDH@rGO showing the elemental composition

**Figure S4:** High resolution TEM and STEM images showing LDH and rGO interfaces with dendritic cell like morphologies

**Figure S5**: (a) Comparison of CV curves of rGO,Ni-Co LDH@rGO and hybrid device (Ni-Co LDH@rGO// rGO) and (b) Charge discharge curves of the fabricated hybrid device

**Figure S6**: Impedance spectra obtained for pristine Ni-Co LDH and Ni-Co LDH@rGO

**Figure S7:** Specific capacitance retention determined from cyclic stability of Ni-Co LDH@rGO

**Figure S8: -** Fabricated hybrid supercapacitor device using solid state gel electrolyte (two devices connected in series) for powering red LED as a function of time.

**Figure S9:** Device capacitance retention obtained for about 24 hrs of continuous charge discharge cycling.

**Table 1:- Comparative of device performance with similar devices reported in literature.**

| **Ref no.** | **Positive electrode//negative electrode** | **Energy density(Wh/kg)** | **Power density (W/kg)** |
| --- | --- | --- | --- |
| 4 | NiCo2O4–MnO2//activated graphene | 9.4 | 175 |
| 5 | Ni(OH)2–graphene//AC | 11.1 | 64 |
| 6 | Co3O4 NSs–rGO //AC | 13.4 | 200 |
| 7 | NiCo2O4//AC | 15.4 | 800 |
| 8 | ZnO@MnO2 NFs//AC | 17 | 393 |
| 9 | Co-Mn LDH//AC | 20.3 | 435 |
| 10 | GO/NiAl-LDH//AC | 21 | 800 |
| 11 | Ni(OH)2@3D Ni//AC | 21.8 | 660 |
| 12 | rGO@NiMn-LDH@NF//AC | 22.5 | 700 |
| 13 | rGO/CoAl-LDH//rGO | 22.6 | 900 |
| 14 | NiCo2O4–rGO//AC | 23.3 | 324 |
| 15 | NixCo1−x LDH–ZTO//AC | 23.7 | 284 |
| 16 | MnO2-CNT//ac | 25 | 500 |
| 17 | Co–Al LDHs–CNTs//AC | 28 | 444 |
| 18 | NiCo2O4@MnO2//AC | 35 | 163 |
|  | Ni-Co LDH@rGO//rGO  This work | 35 | 744 |
